# Does the Removal of Spinal Implants Reduce Back Pain?

**DOI:** 10.14740/jocmr2141w

**Published:** 2015-04-08

**Authors:** Hakan Ak, Ismail Gulsen, Tugay Atalay, Muzaffer Gencer

**Affiliations:** a Department of Neurosurgery, School of Medicine, Bozok University, Yozgat, Turkey; b Department of Neurosurgery, School of Medicine, Yuzuncu Yil University, Van, Turkey; c Department of Anesthesiology, School of Medicine, Bozok University, Yozgat, Turkey

**Keywords:** Spinal instrumentation, Back pain, Visual analog scale, Disk herniation, Vertebra fracture

## Abstract

**Background:**

The importance of the removal of spinal implants is known in the presence of infection. However, the benefits and/or risks of the removal of spinal implant for the management of back pain are not clear.

**Methods:**

In this retrospective study, we aimed to evaluate the beneficial effects of the removal of spinal implants for back pain. Study included 25 patients with thoracolumbar instrumentation.

**Results:**

Seventeen (68%) of them were male. Indications for spinal instrumentation were vertebra fracture (n = 9), iatrogenic instability due to multiple segment laminectomy (n = 12), and instrumentation after recurrent disk herniations (n = 4). Mean visual analog score (VAS) before the removal was 8.08. Mean VAS was 3.36 after the removal. Spinal instruments were removed after the observance of the presence of fusion. All patients were prescribed analgesics and muscle relaxants for 3 weeks before removal. Back pain did not decrease in five (20%) patients in total. Four of them had been instrumented due to recurrent lumbar disk herniation. None of the patients reported the complete relief of pain.

**Conclusion:**

In conclusion, patients should be cautioned that their back pain might not decrease after a successful removal of their instruments.

## Introduction

The application of spinal instrumentation plays a significant role in today’s spine surgery. It is performed for different indications such as vertebra fracture, revision operations, cases of degenerative spondylolisthesis, fusion over multiple levels, scoliosis, stenosis with scoliosis or lateral slip or in cases where iatrogenic instability is created at the time of an operation [1]. The removal of posterior spinal instrumentation is not always an easy and benign application. Its exact indications are still in debate [2]. The removal of spinal instruments may be needed due to late surgical site infection or late operative site pain (Karl Rathjen). The importance of the removal of spinal instruments for the management of infection was shown by different studies [3, 4]. However, there are no clear results about the benefits and risks of removal of instrumentation for pain.

In the present study, we aimed to evaluate whether the removal of spinal instruments decreases back pain or not.

## Materials and Methods

The present study included a retrospective analysis of patients in whom the posterior instrumentations were removed in Teaching and Research Hospitals of Bozok University and Yuzuncu Yil University. Hospital folders of 40 patients were retrospectively searched for demographic data such as age, gender, previous indication for posterior segmental instrumentation, and visual analog score (VAS) before and after removal of spinal instruments. Ten patients in whom removal had been performed due to infection and instrument-related problems such as malposition and/or breakage of the instruments were excluded from the study. Five patients with inadequate data or those who could not be reached were also excluded from the study; thereby 25 patients were enrolled into the study. Pediatric patients were also excluded from the study. All patients whose instruments were removed had been treated with analgesics and muscle relaxants for 3 weeks before removal. Computed tomography images of spine were obtained in all patients with the aim of the observance of the development of the adequate fusion. Spinal instruments were removed in patients with adequate fusion.

## Results

Twenty-five patients were included in the study. Seventeen (68%) of them were male and the remaining were female. The mean age was 44.40 (min. 27 - max. 65, standard deviation: 11.76). The mean VAS was 8.08 (min. 7.0 - max. 9.0) before removal of instruments. The mean VAS was 3.36 (min. 1 - max. 9.0) after removal. The mean decrease in VAS was 4.72 (min. 0 - max. 7) (Table 1).

**Table 1:** Descriptive Statics of the Patients

	N	Minimum	Maximum	Mean	Standard deviation
Age	25	27	65	44.40	11.762
VAS before removal	25	7.00	9.00	8.08	0.640
VAS after removal	25	1.00	9.00	3.36	2.643
Decrease in VAS	25	0.00	7.00	4.72	2.389

Indications for posterior segmental instrumentation were vertebra fracture (n = 9, 36%, group I), iatrogenic instability due to multiple segment laminectomy (n = 12, 48%, group II), and instrumentation after recurrent disk herniations (n = 4, 16%, group III). The mean age was 37.56, 51.50, and 38.50 in groups I, II, and III, respectively. The mean VAS before removal of instruments was 7.8, 7.9, and 9.0 in groups I, II, and III, respectively. The mean VAS was 2.11, 3.08, and 7.0 in groups I, II, and III, respectively after removal of instruments. The mean decrease in VAS was 5.7, 4.83, and 2.0 in groups I, II, and III, respectively. ANOVA showed statistically significant differences between groups in terms of age, VAS before removal, VAS after removal, and decrease in VAS (P = 0.009, 0.003, 0.003, and 0.23, respectively) (Table 2).

**Table 2:** Results of the ANOVA

	Sum of squares	df	Mean square	F	Sig.
Age					
Between groups	1,165,778	2	582,889	5,953	0.009
Within groups	2,154,222	22	97,919		
Total	3,320,000	24			
VAS before removal					
Between groups	4,034	2	2,017	7,644	0.003
Within groups	5,806	22	0.264		
Total	9,840	24			
VAS after removal					
Between groups	67,954	2	33,977	7,490	0.003
Within groups	99,806	22	4,537		
Total	167,760	24			
Decrease in VAS					
Between groups	39,818	2	19,909	4,505	0.023
Within groups	97,222	22	4,419		
Total	137,040	24			

Patients were divided into three groups according to the indications of posterior spinal segmental instrumentation: vertebra fracture (group I), iatrogenic instability due to multiple segment laminectomy (group II), and instrumentation after recurrent disk herniations (group III). There was a statistically significant difference in group I and group II in terms of age (P = 0.01). However, there was no significant difference between other groups in terms of age. Statistically significant differences were observed between groups I - III and groups II - III in terms of VAS before removal (P = 0.04, for each group). When groups were compared in terms of VAS after removal of instruments, a statistically significant difference was observed between groups I - III and groups II - III (P = 0.003 and P = 0.01, respectively). A statistically significant difference was detected only between groups I and III in terms of decrease in VAS (P = 0.018) (Table 3).

**Table 3:** Results of Multiple Comparisons of Groups

Dependent variable	(I) diagnosis	(J) diagnosis	Mean difference (I-J)	Standard error	Sig.	95% confidence interval	
						Lower bound	Upper bound
Age	Group I	Group II	-13.94	4.36	0.011	-24.91	-2.98
		Group III	-0.94	5.94	0.986	-15.88	13.99
	Group II	Group III	13.00	5.71	0.081	-1.35	27.35
VAS before removal	Group I	Group II	-0.027	0.22	0.992	-0.59	0.54
		Group III	-1.11	0.30	0.004	-1.88	-0.33
	Group II	Group III	-1.08	0.296	0.004	-1.82	-0.33
VAS after removal	Group I	Group II	-0.97	0.939	0.563	-3.33	1.38
		Group III	-4.88	1.27	0.003	-8.10	-1.67
	Group II	Group III	-3.91	1.22	0.011	-7.00	-0.82
Decrease in VAS	Group I	Group II	0.94	0.92	0.573	-1.38	3.27
		Group III	3.77	1.26	0.018	0.60	6.95
	Group II	Group III	2.83	1.21	0.072	-0.21	5.88

### Statistical analysis

Quantitative variables were expressed as mean ± standard deviation (SD) or median with range, and were analyzed by Student’s *t*-tests, one-way ANOVA, Mann-Whitney U tests or Kruskal-Wallis tests as appropriate. Statistical analyses were performed using the statistical package SPSS, version 15.0 (SPSS Inc., Chicago IL, USA); a value of P < 0.05 was used to define statistical significance.

## Discussion

The results of the present study indicate that the removal of posterior instruments does not lead to back pain free life especially in patients who have been instrumented after recurrent disk herniations. We saw that in 20% of our patients, no change was observed in their back pains.

Reoperation after posterior segmental instrumentation is not a rare application. Reoperation indications include delayed surgical site infection, breakage of instruments, malposition of instruments, development of adjacent segment disease, implant prominence, and operative site pain [2]. It was reported that the rate of delayed surgical site infection after posterior spinal instrumentation is about 5% [5]. The importance of the removal of the posterior spinal instruments for the management of infection was shown by different studies [3, 4].

The removal of spinal instrument for the pain management is a known application. Reoperation rates for the management of pain range between 8% and 19% [6]. However, there are no clear results about the benefits and risks of removal of instrumentation for pain. The data about this topic mainly come from the studies which include patients who had been operated due to scoliosis. In those studies authors reported the deterioration of curve after removal of instruments in scoliosis patients [7-9]. However, in our study, patients had been operated due to different indications other than scoliosis.

In a similar study, Stavridis et al reported that 12% of their patients (seven of 57) were free of pain. They stated that the 61% of their patients showed some benefit. Authors also reported the occurrence of complication in 8.8% of patients [10]. However, in our study, no complication occurred. However, none of our patients were free of pain. Five patients reported no change in VAS. Three of them had been instrumented after recurrent disk herniation. A possible explanation of the pain mechanism in those patients may be recurrent stripping of back muscles. Alanay et al reported that VAS decrease after implant removal was 50% in a different study [11]. The mean decrease in VAS was 4.72 in all of our patients (Table 4).

**Table 4:** Data of Patients

n	Age	Gender	Prior diagnosis	VAS before removal	VAS after removal	VAS difference
1	35	M	L1 fracture	8	2	6
2	56	M	Stenosis	7	2	5
3	64	M	Stenosis	8	2	6
4	29	M	L2 fracture	9	3	6
5	44	F	Stenosis	8	2	6
6	65	M	L1 fracture	9	3	6
7	28	M	L3 fracture	8	2	6
8	36	F	L2 fracture	7	2	5
9	27	M	L4 fracture	7	2	5
10	55	F	Stenosis	8	3	5
11	65	M	Stenosis	8	2	6
12	49	F	Stenosis	8	2	6
13	38	M	Recurrent disk herniation	9	9	0
14	44	F	Stenosis	8	2	6
15	29	M	L4 fracture	7	1	6
16	33	M	L1 fracture	8	1	7
17	44	F	Stenosis	8	1	7
18	55	F	Stenosis	8	8	0
19	45	M	Stenosis	8	3	5
20	46	M	Stenosis	8	2	6
21	44	M	Recurrent disk herniation	9	2	7
22	56	M	L3 fracture	8	3	5
23	51	F	Stenosis	8	8	0
24	34	M	Recurrent disk herniation	9	8	1
25	38	M	Recurrent disk herniation	9	9	0

In conclusion, the results of the present study and previous studies clearly show that the removal of instruments for the management of back pain does not lead to free of pain in all patients. Because of this reason, patients should be strictly informed that their back pain may sustain in spite of a successful surgery.
